# Effect of prophylactic transcatheter arterial chemoembolization on hepatocellular carcinoma with microvascular invasion after R0 resection. A case-control study

**DOI:** 10.1590/1516-3180.2019.0042.R1.05072019

**Published:** 2020-04-22

**Authors:** Ke-Yue Li, Shuai-Min Zhang, Cheng-Xian Shi, Ke-Li Tang, Jian-Zhao Huang

**Affiliations:** I MD, PhD. Associate Professor of Surgery, Department of Hepatobiliary Surgery, Guizhou Provincial People’s Hospital, Guiyang, Guizhou Province, China.; II MD, MMed. Attending Physician, Department of Hepatobiliary Surgery, Guizhou Provincial People’s Hospital, Guiyang, Guizhou Province, China.; III MD, MMed. Professor of Surgery, Department of Hepatobiliary Surgery, Guizhou Provincial People’s Hospital, Guiyang, Guizhou Province, China.; IV MD, MMed. Professor of Surgery, Department of Hepatobiliary Surgery, Guizhou Provincial People’s Hospital, Guiyang, Guizhou Province, China.; V MD, PhD. Professor of Surgery, Department of Hepatobiliary Surgery, Guizhou Provincial People’s Hospital, Guiyang, Guizhou Province, China.

**Keywords:** Carcinoma, hepatocellular, Chemoembolization, therapeutic, Arteries, Postoperative period, HCC, TACE, MVI

## Abstract

**BACKGROUND:**

Transcatheter arterial chemoembolization (TACE) is thought to prevent recurrence of hepatocellular carcinoma (HCC), but its efficacy is a matter of controversy.

**OBJECTIVES:**

We investigated the effect of preventive TACE on the tumor, nodes, metastasis (TNM) classification in cases of stage II HCC (T2N0M0) after R0 resection.

**DESIGN AND SETTING:**

Case-control study conducted in a tertiary-level public hospital.

**METHODS:**

We analyzed recurrence rates and mortality rates over time for 250 consecutive cases of HCC in TNM classification cases of stage II HCC (T2N0M0) after R0 resection. These cases were divided into patients who underwent TACE (TACE+) and presented microvascular invasion (MVI+; n = 80); TACE+ but did not present MVI (MIV−; n = 100); MVI+ but did not undergo TACE (TACE−, n = 30); and TACE−/MVI− (n = 40).

**RESULTS:**

MVI+ patients in the TACE+ group had significantly lower recurrence rates and mortality rates at one, two and three years than those in the TACE- group (all P < 0.05). Among MVI- patients, the TACE+ group did not have significantly lower recurrence rates and mortality rates at one, two and three years than the TACE- group (all P > 0.05). Regardless of whether TACE was performed or not, MVI− patients had significantly lower recurrence rates and mortality rates at two and three years after their procedures than did MVI+ patients (all P < 0.05).

**CONCLUSION:**

Recurrence rates and mortality rates for MVI+ patients were significantly higher than for MVI− patients, beyond the first year after TACE. Postoperative adjuvant TACE may be beneficial for HCC patients with MVI.

## INTRODUCTION

Hepatocellular carcinoma (HCC) is one of the most common malignancies in the world^1^^,^^2^ and causes around 500,000 deaths every year.^3^ Although hepatectomy and liver transplantation are considered to be curative therapies for HCC,^1^ HCC often relapses after surgery. Transcatheter arterial chemoembolization (TACE) is thought to prevent recurrence, but its efficacy is a matter of controversy.^4^

## OBJECTIVES

To analyze the effect of preventive TACE on recurrence rates and mortality rates among patients with the TNM classification of tumors of patients with stage II HCC (T2N0M0) of HCC who underwent R0 resection. Our hypothesis for this study was that TACE would be equally effective for HCC patients with or without MVI.

## METHODS

### Study design and ethics

In this case-control study, we analyzed recurrence rates and mortality rates among 250 consecutive cases of HCC with TNM classification stage II (T2N0M0) after R0 resection. We compared four groups of patients according to presence of microvascular invasion and use of TACE.

All the patients gave their informed consent to participation in this study. The study was approved by our institution’s ethics committee on January 4, 2005, under the approval protocol number 2005006.

### Patients

We followed up all the 250 consecutive patients with HCC who underwent R0 resection between January 2005 and December 2014, over a 36-month period after their surgeries. All of these patients were treated in the Department of Hepatobiliary Surgery, Guizhou Provincial People’s Hospital. The inclusion criteria were as follows:

1. Age more than 16 years and less than 65 years;
2. Histopathological classification of high/medium differentiation;
3. TNM classification as stage II (T2N0M0) for HCC;
4. Treatment by means of extended resection of the tumor, with resection margins of 2 cm;
5. Liver function: Child-Pugh score of not more than 9 points.

The exclusion criteria were as follows:

1. Presence of other serious life-threatening diseases, such as severe coronary heart disease, another malignant tumor, etc.;
2. Evidence of liver abscess, abdominal infection, biliary fistula or intraperitoneal hemorrhage;
3. Pregnancy.

We divided the cohort into four groups: Group 1, who underwent TACE (TACE+) and presented microvascular invasion (MVI+; n = 80); Group 2, who were TACE+ but did not present MVI (MIV−; n = 100); Group 3, who were MVI+ but did not undergo TACE (TACE−, n = 30); and Group 4, who were TACE−/MVI− (n = 40).

### TACE

Patients who underwent TACE did so within one to two months after their hepatectomies (Table 1). A hepatic arterial catheter was placed into the proper hepatic artery through the femoral artery using the Seldinger technique, and TACE was performed for the entire remnant liver. Hepatic angiography, computed tomography (CT) angiography, or both, were performed to detect any obvious tumor stains in the remnant liver.

**Table 1.** Demographic and clinical data of the four patient groups

	Group 1	Group 2	Group 3	Group 4	P
*N*	80 (M/F: 43/37)	100 (M/F: 51/49)	30 (M/F: 17/13)	40 (M/F: 21/19)	
Age (years)	48.62 ± 11.32	46.63 ± 11.61	45.45 ± 11.51	47.55 ± 11.55	> 0.05
Complications (%)	0.32 ± 0.13	0.35 ± 0.15	0.33 ± 0.12	0.34 ± 0.14	> 0.05
Liver function:					
Child-Pugh score	7.5 ± 1.4	7.3 ± 1.5	7.4 ± 1.3	7.4 ± 1.5	> 0.05

The P-values refer to the comparisons of age, complications and liver function Child-Pugh score in each group.

MVI = microvascular invasion; TACE = transcatheter arterial chemoembolization.

Group 1: underwent TACE and had MVI; Group 2: underwent TACE but did not have MVI; Group 3: had MVI but did not undergo TACE; Group 4: did not undergo TACE or have MVI.

The TACE procedure was a “sandwich” method, in which iodide oil (1 ml to 2 ml) was injected before and after administering chemotherapy. The chemotherapy regimen included fluorouracil, a platin (cisplatin or carboplatin) and adriamycin (doxorubicin or epirubicin). The dosages of fluorouracil, platin and adriamycin were determined according to body surface area and underlying liver function. All patients in this study who underwent prophylactic TACE received only one prophylactic TACE treatment, within two months after their surgery.

In order to make comparisons and avoid bias, we selected the cases with similar age (16 to 65 years), tumor differentiation (high/medium differentiation), tumor stage (T2N0M0) and Child-Pugh score for liver function (not more than nine points) and the cases with fewer complications (cases without liver abscess, abdominal infection, biliary fistula or intraperitoneal hemorrhage, etc.) and clean cutting edges (with resection margins of 2 cm). The aim of making this selection was to minimize other possible factors. We collected data mainly from the medical records (the period of time that was considered for data collection was from January 2005 to December 2017). In a very small number of cases, we collected data through patient follow-up.

### Statistical analysis

We analyzed recurrence rates and mortality rates at one, two and three years after the procedures that were performed on these patients. Statistical analyses were performed using SPSS 16.0 for Windows (SPSS Inc., Chicago, IL, USA). The differences between groups of data were analyzed by means of the chi-square test (two-tailed). P-values < 0.05 were considered statistically significant.

## RESULTS

During the study period, 280 patients were admitted to our hospital service presenting HCC, and 30 patients were excluded. The patients included comprised 131 males and 119 females. Their average age was 48.01 years (range: 16-65 years). The recurrence rates and mortality rates for each patient group, over each time period, are shown in Tables 2**,**3**,**4**and**5.

**Table 2.** Recurrence rates among patients who did or did not undergo TACE

	Postoperative time	With TACE	Without TACE	P-value
With MVI	12 months	20/80 (25.0%)	14/30 (46.7%)	0.029
	24 months	35/80 (43.8%)	20/30 (66.7%)	0.032
	36 months	44/80 (55.0%)	23/30 (76.7%)	0.038
Without MVI	12 months	20/100 (20.0%)	9/40 (22.5%)	0.742
	24 months	29/100 (29.0%)	15/40 (37.5%)	0.328
	36 months	40/100 (40.0%)	21/40 (52.5%)	0.178

MVI = microvascular invasion; TACE = transcatheter arterial chemoembolization.

**Table 3.** Mortality rates among patients who did or did not undergo TACE

	Postoperative time	With TACE	Without TACE	P
With MVI	12 months	17/80 (21.2%)	13/30 (43.3%)	0.021
	24 months	32/80 (40.0%)	19/30 (63.3%)	0.029
	36 months	43/80 (53.8%)	23/30 (76.7%)	0.029
Without MVI	12 months	17/100 (17.0%)	7/40 (17.5%)	0.943
	24 months	26/100 (26.0%)	11/40 (27.5%)	0.856
	36 months	35/100 (35.0%)	18/40 (45.0%)	0.270

MVI = microvascular invasion; TACE = transcatheter arterial chemoembolization.

**Table 4.** Recurrence rates among patients who presented with or without MVI

	Postoperative time	With MVI	Without MVI	P
With TACE	12 months	20/80 (25%)	20/100 (20.0%)	0.423
	24 months	35/80 (43.8%)	29/100 (29.0%)	0.040
	36 months	44/80 (55.0%)	40/100 (40.0%)	0.000
Without TACE	12 months	14/30 (46.7%)	9/40 (22.5%)	0.033
	24 months	20/30 (66.7%)	15/40 (37.5%)	0.016
	36 months	23/30 (76.7%)	21/40 (52.5%)	0.038

MVI = microvascular invasion; TACE = transcatheter arterial chemoembolization.

**Table 5.** Mortality rates among patients who presented with or without MVI

	Postoperative time	With MVI	Without MVI	P
With TACE	12 months	17/80 (21.2%)	17/100 (17.0%)	0.469
	24 months	32/80 (40.0%)	26/100 (26.0%)	0.046
	36 months	43/80 (53.8%)	35/100 (35.0%)	0.012
Without TACE	12 months	13/30 (43.3%)	7/40 (17.5%)	0.018
	24 months	19/30 (63.3%)	11/40 (27.5%)	0.003
	36 months	23/30 (76.7%)	18/40 (45.0%)	0.008

MVI = microvascular invasion; TACE = transcatheter arterial chemoembolization.

Tables 2**and**3 show that, among MVI+ patients, those who underwent TACE (TACE+ group) had significantly lower recurrence rates and mortality rates at one, two and three years after their procedures (all P < 0.05) than did those who did not undergo this procedure (TACE- group). The recurrence rates and mortality rates among the MVI- patients tended to be lower at one, two and three years for the TACE+ group, but not significantly so (all P > 0.05).

Tables 4**and**5 show that, among both TACE+ and TACE− patients, those who were MVI− had significantly lower recurrence rates and mortality rates at two and three years than did those who were MVI+ (all P < 0.05).

## DISCUSSION

Although preventive TACE has become a common post-surgical treatment for HCC,^4^^,^^5^ its efficacy is still a matter of controversy. Support for TACE is based on the fact that compressing a tumor during surgery may lead to its spread. Postoperative TACE helps to clear up any proliferating, remnant or difficult-to-find tumor cells, and thus reduce early recurrence rates.^5^^,^^6^

A meta-analysis on four randomized controlled trials and three non-randomized controlled trials concluded that postoperative adjuvant TACE improves survival rates at two years and three years after resection.^7^ The basis for opposing the use of TACE is that TACE can inhibit patients’ immune systems, thereby contributing to tumor recurrence and metastasis.^8^^,^^9^ Our results showed that among MVI− patients, TACE+ patients tended to have lower recurrence rates and mortality rates at one, two and three years, but not significantly so (P > 0.05), which indicates that preventive TACE cannot benefit MVI− patients.

The Milan criteria classify MVI as an independent risk factor for HCC,^10^ and its presence in the hepatic or portal veins or the bile duct is an accurate predictor of recurrence risk and overall survival in patients with HCC after R0 liver resection and transplantation.^11^^,^^12^^,^^13^^,^^14^ Postoperative adjuvant TACE may be beneficial for HCC patients with MVI.^15^

## CONCLUSIONS

The recurrence and mortality rates among MVI+ patients were significantly higher than those of MVI− patients, beyond the first year after TACE (P < 0.05).

Thus, MVI+ patients may benefit from timely administration of postoperative adjuvant TACE if this is done within one to two months after R0 resection of HCC.
